# Robust Null Broadening Beamforming Based on Covariance Matrix Reconstruction via Virtual Interference Sources

**DOI:** 10.3390/s20071865

**Published:** 2020-03-27

**Authors:** Jian Yang, Jian Lu, Xinxin Liu, Guisheng Liao

**Affiliations:** 1 School of Electronic Engineering, Xidian University, Xi’an 710071, China; 2 School of Engineering, Rocket Force University of Engineering, Xi’an 710025, China

**Keywords:** covariance matrix reconstruction, null broadening, virtual interference sources, beamforming

## Abstract

When jammers move rapidly or an antenna platform travels at high speed, interference signals may move out of the null width in the array beampattern. Consequently, the interference suppression performance can be significantly degraded. To solve this problem, both the null broadening technique and robust adaptive beamforming are considered in this paper. A novel null broadening beamforming method based on reconstruction of the interference-plus-noise covariance (INC) matrix is proposed, in order to broaden the null width and offset the motion of the interfering signals. In the moving case, a single interference signal can have multiple directions of arrival, which is equivalent to the existence of multiple interference sources. In the reconstruction of the INC matrix, several virtual interference sources are set up around each of the actual jammers, such that the nulls can be broadened. Based on the reconstructed INC and signal-plus-noise covariance (SNC) matrices, the steering vector of the desired signal can be obtained by solving a new convex optimization problem. Simulation results show that the proposed beamformer can effectively broaden the null width and deepen the null depth, and its performance in interference cancellation is robust against fast-moving jammers or array platform motion. Furthermore, the null depth can be controlled by adjusting the power parameters in the reconstruction process and, if the direction of interference motion is known, the virtual interference sources can be set to achieve better performance.

## 1. Introduction

As antenna arrays have been applied to radar, sonar, radio astronomy, medical imaging, and in other areas, beamforming is widely used for interference suppression, target detection, and direction of arrival (DOA) estimation [1,2]. The purpose of adaptive beamforming is to enhance the desired signal while simultaneously suppressing interference in the spatial domain [3]. Under mismatches, such as wavefront distortion, array calibration errors, look direction errors, and incoherent local scattering, traditional beamformers suffer from severe performance degradation [4]. To address this problem, various robust adaptive beamformers have been proposed. In recent years, due to its good robustness against mismatches, a novel robust adaptive beamforming method based on covariance matrix reconstruction and steering vector estimation has attracted much attention [5,6,7,8,9]. However, in the presence of fast-moving interference sources or antenna platform movement, such as that which occurs in highly dynamic conditions, the directions of arrival (DOAs) of the interference signals may range over multiple resolution units and the beamforming weights cannot be updated in real time, which will cause the interference to move out of the nulls and decrease the anti-interference capacity of adaptive beamforming techniques. An alternative method is to keep the interference in the nulls by broadening the null widths; that is, a wider attenuation is formed in the region where the interference may appear, in order to effectively suppress the interference direction perturbation. Some research has been carried out in the area of null broadening techniques, including virtual interference sources, derivative constraints, and projection transformations.

Therefore, null broadening techniques can provide effective and robust approaches to overcoming this problem. In References [10,11,12,13,14], these approaches were based on the covariance matrix tapering and effectively broadened the null width. However, this technique decreases the array gain and the depth may become shallower [15]. Semidefinite programming was introduced to improve the performance of adaptive null broadening beamforming in References [16,17]. Moreover, Qian et al. [18] first imposed nulls toward the regions of nonstationary interference via the reconstruction of the interference-plus-noise covariance matrix, and a similarity constraint was enforced at the design stage to restrict the shape of the beam pattern. Then, the adaptive weight vector could be computed via maximizing a new signal-to-interference-plus-noise ratio (SINR) criterion subject to the similarity constraint. In mathematics, the optimisation problem was a nonconvex fractional quadratically constrained quadratic programming (QCQP) problem, which was converted into a convex optimisation problem using semidefinite programming techniques. In Reference [19], by combining with white-noise constraint adaptive processing, the null widening technique could provide effective nulling under snapshot-deficient conditions. Nevertheless, compared to the previous methods, these approaches only result in a small improvement in performance. A new null broadening beamformer based on projection and diagonal loading has been proposed in Reference [20]. Although this approach successfully broadens the null width, its shortcoming is shallower nulls. In Reference [21], by means of the covariance matrix of the auxiliary elements, a null broadening method was realized based on the sidelobe canceller. This approach demonstrated good performance in practical applications, but has increased hardware complexity. In References [22,23], an adaptive null broadening technique based on reconstruction of the covariance matrix was proposed. However, these approaches have poor robustness against various mismatches in the antenna array. Ors et al. [24] proposed first- and second-order iterative null broadening beamforming, and illustrated that the second order required fewer iterations compared to the first order. Nevertheless, the robustness of the beamforming was not considered either. Xiao et al. [25] proposed a method based on optimization algorithm and neural networks to the complicated wide null problem, and the performance of the data-based general regression neural network wide nulling model was demonstrated by numerical experiment. In Reference [26], Z. Liu et al. proposed a computationally efficient null widening method for sidelobe canceller, which is a covariance matrix taper-based method and puts fictitious interferers into snapshots to broaden the sharp null. Based on Mailloux’s methodology, the covariance matrix and cross-correlation vector were tapered via random disturbance. Compared with the existing methods, the method required much less computation, but its performance is similar. However, the performance of the proposed method in the presence of array errors needs to be further studied. In order to solve the problem that the performance of existing null broadening beamforming will decrease when array geometry errors are present, Zhao et al. [27] proposed a null broadening beamforming only against array geometry errors, which was not applied against other errors, such as unknown array steering vector errors, and incoherent local scattering errors. Yang et al. [28] proposed robust wideband adaptive beamforming with null broadening and constant beamwidth via adding virtual interferers around original interferers. For that algorithm, the amount of virtual interference was an important parameter for the taper matrix, but it was difficult to determine and the method of adding virtual interferers was relatively complex. The interference-plus-noise covariance matrix was reconstructed with the simplified power spectral density function and broadened notches corresponding to the time-varying DOAs of the moving interference signals [29]. This method could impose nulls towards the regions of the moving interference signals and obtain lower sidelobes in the beampattern. However, except for DOA mismatch, its robustness against other array errors needs to be further verified and improved. In addition, Wang et al. [30] and Cong et al. [31] proposed Null broadening beamforming methods for anti-jamming in GNSS receivers. However, for their methods, the interferers were assumed to be in a certain distribution, for example, Gauss or trigonometric distribution, which was often not satisfied.

In this paper, we combine the null broadening technique with robust adaptive beamforming and propose a novel adaptive null broadening beamformer based on covariance matrix reconstruction. First, a new interference-plus-noise covariance (INC) matrix is reconstructed by placing several virtual interference sources around each of the actual jammers. Then, the signal-plus-noise covariance (SNC) matrix is reconstructed using the Capon spatial spectrum [32]. Next, the steering vector of the desired signal can be estimated by solving a new convex optimization problem. Finally, based on the reconstructed INC matrix and the estimated steering vector of the desired signal, the weight vector can be obtained according to the minimum variance distortionless response (MVDR) beamformer. Simulation results demonstrate that the robust adaptive beamformer can effectively broaden the null width and has good performance.

The main contributions of this paper can be summarized as follows:

1) Compared with other existing null broadening beamforming techniques, we propose an algorithm based on INC matrix reconstruction by setting up several virtual interference sources around the actual jammers directly in the Capon spatial spectrum, which can simultaneously broaden the nulls and eliminate the self-null phenomenon of the desired signal. Furthermore, the null depth and width can be controlled by setting the parameters of the virtual interference sources.

2) Based on the reconstructed INC and SNC matrices, we design a convex optimization problem to obtain the steering vector of the desired signal, in order to further improve the robustness of algorithm.

The rest of this paper is organized as follows. In Section 2, the signal model, including the antenna array and the received signal, is given. In Section 3, the proposed algorithm is introduced, which consists of INC matrix reconstruction, SNC matrix reconstruction, and steering vector estimation. Section 4 presents the simulation setup and results. Finally, Section 5 gives some concluding remarks.

## 2. Problem Background

Assume that a uniform linear array (ULA) comprised of *N* omni-directional sensors receives M+1 far-field narrowband source signals. The first signal is the desired signal, impinging upon the array from the direction $\theta_{s}$, while the remaining *M* interfering signals come from directions $\theta_{1},\theta_{2},\ldots ,\theta_{M}$, respectively. The array sample data at the $k^{\mathrm{th}}$ snapshot can be modeled as

(1) $$\begin{array}{cc} x(k) & =x_{s}\left(k\right)+x_{\mathrm{int}}\left(k\right)+n\left(k\right)\\ \; & =x_{s}\left(k\right)a\left(\theta_{s}\right)+\sum_{m=1}^{M}x_{\mathrm{int}}\left(k\right)a\left(\theta_{m}\right)+n\left(k\right), \end{array}$$

where $a(\theta_{s})$ is the steering vector of the desired signal, $a(\theta_{m})$ is the steering vector of the mth interference signal, and $x_{s}\left(k\right)=x_{s}\left(k\right)a\left(\theta_{s}\right)$, $x_{\mathrm{int}}\left(k\right)=\sum_{m=1}^{M}x_{\mathrm{int}}\left(k\right)a\left(\theta_{m}\right)$, and n(k) denote the desired signal, interference signals, and additive white Gaussian noise vectors, respectively. In addition, we assume that they are statistically independent from each other. The steering vector of the ULA can be written as

(2) $$a\left(\theta \right)={\left[1,e^{j2\pi \sin\theta \frac{d}{\lambda }},\ldots ,e^{j2\pi \sin\theta \frac{(N-1)d}{\lambda }}\right]}^{T},$$

where ${\left(\cdot \right)}^{T}$ is the transpose product, *d* is the sensor interval, λ is the wavelength, and θ is the DOA of the impinging signal. The theoretical covariance matrix of the array data can be obtained by

(3) $$R_{x}=E\left\{x\left(k\right)x^{H}\left(k\right)\right\}=\sum_{m=0}^{M}\delta_{m}^{2}a\left(\theta_{m}\right)a^{H}\left(\theta_{m}\right)+\delta_{n}^{2}I,$$

where ${\left(\cdot \right)}^{H}$ is the Hermitian transpose, E(·) is the mathematical expectation, ${\left\{\sigma_{m}^{2}\right\}}_{m=0}^{M}$ are the powers of the impinging signals, $\sigma_{n}^{2}$ is the noise power, and I is the N×N identity matrix. In practice, $R_{x}$ is usually unavailable, and is often replaced by the sample covariance matrix

(4) $${\tilde{R}}_{x}=\frac{1}{K}\sum_{k=1}^{K}x\left(k\right)x^{H}\left(k\right),$$

where *K* is the number of snapshots. The output of the adaptive beamformer is expressed as

(5) $$y\left(k\right)=w^{H}x\left(k\right),$$

where w is the N×1 weight vector. With the MVDR criterion, the optimal beamformer can be obtained by solving the following problem

(6) $$\min_{w}w^{H}R_{\mathrm{int}+n}w\;\;\;\;\mathrm{subject}\;\mathrm{to}\;\;\;w^{H}a\left(\theta_{s}\right)=1,$$

where $R_{\mathrm{int}+n}$ denotes the INC matrix. Therefore, the optimal weight vector can be expressed as

(7) $$w_{opt}=\frac{R_{\mathrm{int}+n}^{-1}a\left(\theta_{s}\right)}{a^{H}\left(\theta_{s}\right)R_{\mathrm{int}+n}^{-1}a\left(\theta_{s}\right)}.$$

However, in practice, the INC matrix cannot be obtained directly, and the sample covariance matrix ${\tilde{R}}_{x}$ is usually used to replace the INC matrix, which may cause the self-nulling phenomenon of the desired signal and result in significant performance deterioration, especially with high SNR [4].

## 3. The Proposed Algorithm

In this section, a new adaptive null broadening beamforming method is proposed. The main idea is to reconstruct a new INC matrix by utilizing virtual interference sources and estimate the steering vector of the desired signal by solving a new optimization problem. As the sample covariance matrix is usually inevitably mixed into the desired signal, which can cause the self-null phenomenon with a high signal-to-noise ratio (SNR) and result in significant performance deterioration, it is necessary to remove the desired signal component from the sample covariance matrix and reconstruct the INC matrix. On the other hand, to avoid convergence to the DOA of the interfering signal, the SNC matrix is also reconstructed to effectively improve the pointing accuracy of the estimated steering vector. Moreover, the method used to reconstruct the INC matrix is also used for SNC matrix reconstruction. Therefore, this section is divided into three parts: The first part discusses the INC matrix reconstruction, the second reconstructs the SNC matrix, and the last estimates the steering vector of the desired signal based on the INC and SNC matrices. Furthermore, the complete algorithmic flow is detailed in the third part.

### 3.1. Inc Matrix Reconstruction

The Capon spatial spectrum [32] estimator is

(8) $$P\left(\theta \right)=\frac{1}{a^{H}\left(\theta \right){\tilde{R}}_{x}^{-1}a\left(\theta \right)}.$$

Using the Capon spatial spectrum (8), an INC matrix reconstruction approach has been proposed [4,5], which uses the following

(9) $${\tilde{R}}_{i+n}=\int_{\bar{\Theta }}P(\theta )a\left(\theta \right)a^{H}\left(\theta \right)d\theta =\int_{\bar{\Theta }}\frac{a\left(\theta \right)a^{H}\left(\theta \right)}{a^{H}\left(\theta \right){\tilde{R}}_{x}^{-1}a\left(\theta \right)}d\theta ,$$

where $\bar{\Theta }$ denotes the angular region excluding the desired signal region Θ, $\Theta \cup \bar{\Theta }$ covers the whole spatial domain, and $\Theta \cap \bar{\Theta }$ is empty. Considering the structured feature of the integral, the reconstructed INC matrix can be approximately calculated by a discrete sum in the discrete time domain as

(10) R˜i+n≈∑k=1Θ¯Θ¯ΔθΔθP(θk)a(θk)aH(θk)Δθ,

where Δθ is the angle interval, which should be small.

In the INC matrix reconstruction, the range of virtual interference sources can be determined according to the motion of the antenna platform or jammers. Assume that *M* regions of virtual interference sources are located in $\left[\vartheta_{1}-\beta_{11},\vartheta_{1}+\beta_{12}\right]$, …,$\left[\vartheta_{M}-\beta_{M1},\vartheta_{M}+\beta_{M2}\right]$, respectively, and the spatial spectra are correspondingly set as $\kappa_{1}P\left(\vartheta_{1}\right),$…$,\kappa_{M}P\left(\vartheta_{M}\right)$. In the array beampattern, the numerical pairs $\left(\beta_{11},\beta_{12}\right)$, …, $\left(\beta_{M1},\beta_{M2}\right)$ determine the regions of null broadening (which may be different in practice) and the respective null widths are $\beta_{12}+\beta_{11}$, …, $\beta_{M2}+\beta_{M1}$. In addition, $\beta_{ij}\;\left(1⩽i⩽M,1⩽j⩽2\right)$ is usually greater than zero. It should be noted that the relative motion between the interference sources and the antenna array is known, which usually could be obtained in advance by DOA estimated techniques and the inertial measurement system, the virtual interference sources can be set up according to the incoming direction of the interference signals to achieve better performance in interference suppression. In particular, it can be realized by controlling these parameters $\beta_{ij}$. The parameters $\kappa_{1},\ldots ,\kappa_{M}$ determine the depth of the nulls, which is closely related to the performance of interference suppression. Meanwhile, $\kappa_{m}$ will cause the mainlobe to widen and the sidelobe to be higher in the beampattern, which are considered to be negative effects. The parameters $\kappa_{m}$ are introduced to better suppress interference, where $\kappa_{m}$ can be determined by the value of $P(\vartheta_{m})$. Specifically, the vector composed of $P\left(\vartheta_{m}\right),\left(1⩽m⩽M\right)$ is arranged in ascending order; that is, its first and last elements, respectively, correspond to $\kappa_{1}=1$ and $\kappa_{M}=10$, and the remaining values of $\kappa_{m}$ can be obtained by linear fitting. Therefore, by (10), the INC matrix based on virtual interference sources in the Capon spatial spectrum can be reconstructed as

(11) R^i+n=∑i=1Θ¯1Θ¯1ΔθΔθκip(θi)a(θi)aH(θi)Δθ+∑k=1Θ¯2Θ¯2ΔθΔθp(θk)a(θk)aH(θk)Δθ,

where ${\bar{\Theta }}_{1}\;=\;\left[\vartheta_{1}-\beta_{11},\vartheta_{1}+\beta_{12}\right]\cup \left[\vartheta_{2}-\beta_{21},\vartheta_{2}+\beta_{22}\right]\cup \ldots \cup \left[\vartheta_{M}-\beta_{M1},\vartheta_{M}+\beta_{M2}\right]$ denotes the angular sector including all interference and virtual interference signals, and ${\bar{\Theta }}_{2}$ denotes the angular sector excluding the desired, interference and virtual interference signals. $\bar{\Theta }\;=\;{\bar{\Theta }}_{1}\cup {\bar{\Theta }}_{2}$, ${\bar{\Theta }}_{1}\cap {\bar{\Theta }}_{2}$ is empty.

### 3.2. Snc Matrix Reconstruction

In order to accurately estimate the steering vector of the desired signal, the SNC matrix can be reconstructed by the Capon spectrum. In the same manner as (9), we can reconstruct the SNC matrix as

(12) $${\tilde{R}}_{s+n}=\int_{{\bar{\Theta }}^{\prime }}\frac{a\left(\theta \right)a^{H}\left(\theta \right)}{a^{H}\left(\theta \right){\tilde{R}}_{x}^{-1}a\left(\theta \right)}d\theta ,$$

where ${\bar{\Theta }}^{\prime }$ denotes the angular region excluding the interference signal regions $\Theta^{\prime }$, $\Theta^{\prime }\cup {\bar{\Theta }}^{\prime }$ covers the whole spatial domain, and $\Theta^{\prime }\cap {\bar{\Theta }}^{\prime }$ is empty.

### 3.3. Desired Signal Steering Vector Estimation

Due to many mismatches, such as gain and phase perturbations, incoherent local scattering, signal look direction error, array geometry errors, the steering vector of the desired signal is often inaccurate. To obtain the optimal weight vector, we introduce the convex theory and obtain the accurate steering vector by solving the optimization problem. In the presence of steering vector mismatches, the mismatch vector $a-a(\theta_{s})$ with a denoting the actual desired signal steering vector can be further decomposed into two components, that is, $a-a(\theta_{s})=e_{⊥}+e_{\left|\right|}$. One denoted by $e_{⊥}$ is orthogonal to $a(\theta_{s})$, and the other denoted by $e_{\left|\right|}$ is parallel to $a(\theta_{s})$, and $e_{\left|\right|}$ does not affect the beamforming quality and does not impact the output SINR [5]. Therefore, a new constraint can be established as

(13) $${\left(\hat{a}-a\left(\theta_{s}\right)\right)}^{H}a\left(\theta_{s}\right)=0,$$

where $\hat{a}=a(\theta_{s})+e_{⊥}$. Furthermore, the spatial power estimate must satisfy

(14) $$\frac{1}{{\hat{a}}^{H}{\tilde{R}}_{s+n}^{-1}\hat{a}}⩾\frac{1}{a^{H}\left(\theta_{s}\right){\tilde{R}}_{s+n}^{-1}a\left(\theta_{s}\right)}.$$

Therefore, the optimization problem can be written as

(15) $$\begin{array}{cc} \; & \min_{\hat{a}}\;\;{\hat{a}}^{H}{\tilde{R}}_{x}^{-1}\hat{a},\\ \; & \mathrm{subject}\;\mathrm{to}\;\;\;\;{\left(\hat{a}-a\left(\theta_{s}\right)\right)}^{H}a\left(\theta_{s}\right)=0,\\ \; & \;\;\;\;\;\;\;\;\;\;\;\;\;\;\;\;\;\;{\hat{a}}^{H}{\tilde{R}}_{s+n}^{-1}\hat{a}⩽a^{H}\left(\theta_{s}\right){\tilde{R}}_{s+n}^{-1}a\left(\theta_{s}\right). \end{array}$$

By solving the optimization problem, the desired signal steering vector can be obtained. Set $\hat{a}=x+jy$, $a(\theta_{s})=c+jb$, and $a^{H}\left(\theta_{s}\right){\tilde{R}}_{s+n}^{-1}a\left(\theta_{s}\right)=\Phi_{1}$, then the objective function can be further written as

(16) $$\left(x^{T}-jy^{T}\right){\tilde{R}}_{x}^{-1}\left(x+jy\right)=x^{T}{\tilde{R}}_{x}^{-1}x+y^{T}{\tilde{R}}_{x}^{-1}y.$$

The first constraint can be expressed as

(17) $$\left\{\begin{array}{c} c^{T}x+b^{T}y-c^{T}c-b^{T}b=0\\ b^{T}x-c^{T}y=0 \end{array}\right.,$$

and the second constraint is equivalent to the following inequality,

(18) $$x^{T}{\tilde{R}}_{s+n}^{-1}x+y^{T}{\tilde{R}}_{s+n}^{-1}y-\Phi_{1}\leq 0.$$

According to (16)–(18), the optimization problem can be further written as

(19) $$\begin{array}{cc} \; & \min_{x,y}x^{T}{\hat{R}}_{x}^{-1}x+y^{T}{\hat{R}}_{x}^{-1}y\\ \mathrm{subject}\;\mathrm{to}\;\;\;\; & c^{T}x+b^{T}y-c^{T}c-b^{T}b=0\\ \; & b^{T}x-c^{T}y=0\\ \; & x^{T}{\tilde{R}}_{s+n}^{-1}x+y^{T}{\tilde{R}}_{s+n}^{-1}y-\Phi_{1}\leq 0 \end{array}.$$

With Karush–Kuhn–Tucker (KKT) conditions [33], the Lagrange function can be expressed as

(20) $$\begin{array}{cc} L(x,y,\lambda_{1},\lambda_{2},\lambda_{3}) & =x^{T}{\tilde{R}}_{x}^{-1}x+y^{T}{\tilde{R}}_{x}^{-1}y+\lambda_{1}\left(c^{T}x+b^{T}y-c^{T}c-b^{T}b\right)\\ \; & +\lambda_{2}\left(b^{T}x-c^{T}y\right)+\lambda_{3}\left(x^{T}{\tilde{R}}_{s+n}^{-1}x+y^{T}{\tilde{R}}_{s+n}^{-1}y-\Phi_{1}\right) \end{array}.$$

Hence, we can obtain

(21) $$\begin{array}{cc} \; & \nabla_{x}L\left(x,y,\lambda_{1},\lambda_{2},\lambda_{3}\right)=2{\tilde{R}}_{x}^{-1}x+\lambda_{1}c+\lambda_{2}b+2\lambda_{3}{\tilde{R}}_{s+n}^{-1}x=0\\ \; & \nabla_{y}L\left(x,y,\lambda_{1},\lambda_{2},\lambda_{3}\right)=2{\tilde{R}}_{x}^{-1}y+\lambda_{1}b-\lambda_{2}c+2\lambda_{3}{\tilde{R}}_{s+n}^{-1}y=0\\ \; & \nabla_{\lambda_{1}}L\left(x,y,\lambda_{1},\lambda_{2},\lambda_{3}\right)=c^{T}x+b^{T}y-c^{T}c-b^{T}b=0\\ \; & \nabla_{\lambda_{2}}L\left(x,y,\lambda_{1},\lambda_{2},\lambda_{3}\right)=b^{T}x-c^{T}y=0\\ \; & \nabla_{\lambda_{3}}L\left(x,y,\lambda_{1},\lambda_{2},\lambda_{3}\right)=x^{T}{\tilde{R}}_{s+n}^{-1}x+y^{T}{\tilde{R}}_{s+n}^{-1}y-\Phi_{1}=0 \end{array}.$$

By solving (21), we can obtain x and y, following which the steering vector of the desired signal can be obtained as $\hat{a}=x+jy$. On the other hand, the optimization problem (15) can be easily solved using convex optimization software, such as CVX [34]. To date, both the INC matrix and the steering vector of the desired signal have been obtained. From (7), (11) and (15), the weight vector of the adaptive null broadening beamformer can be written as

**Figure 1:**
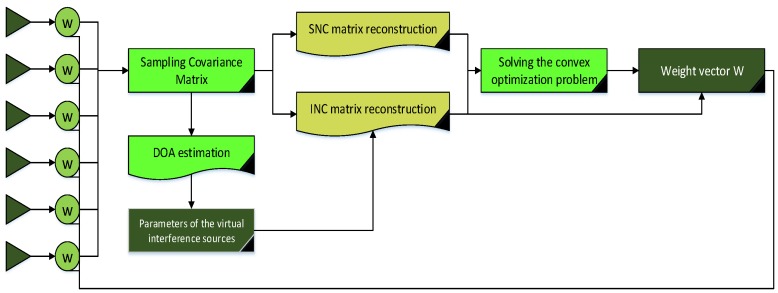
Flow of the proposed beamformer algorithm.

As shown in Figure 1, the proposed approach can be illustrated step-by-step. Therefore, the proposed adaptive null broadening beamforming technique based on covariance matrix reconstruction via virtual interference sources can be summarized as follows:

Step 1. Calculate the Capon spatial spectrum (8) and search for the peaks $\left(\vartheta_{1},\ldots ,\vartheta_{M}\right)$ from the interfering signal region;

Step 2. Reconstruct the new INC matrix (11) based on the virtual interference sources, where $\beta_{ij}$ controls the null width and $\kappa_{i}$ controls the null depth;

Step 3. Estimate the steering vector $\hat{a}$ by solving the optimization problem (15); and

Step 4. Calculate the weight vector $w_{opt-null}$ (22).

In the proposed algorithm, the computational complexity is dominated by solving the optimization problem, which can be efficiently solved using an interior point method with a computational complexity cost of $O(N^{3.5})$. The robust adaptive beamforming algorithm in Reference [5] has a complexity of $O(N^{3.5})$, due to the use of a quadratically constrained quadratic programming problem. Therefore, from a computational complexity point of view, the cost of the proposed algorithm is similar to that of the robust adaptive beamforming algorithm in Reference [5], and is comparable with that of other robust beamforming algorithms [5,9].

(22) $$w_{opt-null}=\frac{{\hat{R}}_{i+n}^{-1}\hat{a}}{{\hat{a}}^{H}{\hat{R}}_{i+n}^{-1}\hat{a}}.$$

## 4. Simulation Results

In our simulations, a ULA consisting of N=10 omni-directional sensors with an inter-sensor spacing of half a wavelength was considered. We assumed that the additive noise obeys a Gaussian distribution with unit covariance. In the simulations, the proposed algorithm was compared with the following methods: (i) the robust adaptive beamformer (Rec-ISVPE) of Reference [5], (ii) the Laplace-based null broadening algorithm of Reference [10], (iii) the Mailloux-based null broadening algorithm of Reference [11], (iv) the improved null broadening beamforming based on covariance matrix reconstruction (Rec-MVDR) of Reference [23], and (v) the analytical approach to null broadening (AANB) of Reference [15]. Among these methods, methods (i) and (iv) use INC matrix reconstruction, while the others are null broadening beamforming algorithms. For all tested beamformers, to effectively verify the performance of null broadening beamformers, the width of nulls is set to $4^{\circ }$, and the control parameter *b* of Rec-MVDR is set to 1.2. The optimal weight vector $w_{opt}$ is obtained by (7), which is calculated from the exact INCM and the actual desired signal steering vector. Then, the output optimal SINR can be obtained by SINRopt=σs2woptHa(θs)σs2woptHa(θs)woptHRint+nwoptwoptHRint+nwopt, where $\sigma_{s}^{2}$ denotes the desired signal power [5,35]. For each scenario (except for that in Section 4.1), 200 Monte Carlo runs were performed.

### 4.1. Performance of Null Broadening

In the first example, the beampatterns of null broadening were examined. Assumed that the desired signal with fixed 5 dB SNR comes from the direction $\theta_{s}=20^{\circ }$, and two interfering signals impinge upon the array antenna from the directions $\theta_{1}=-30^{\circ }$ and $\theta_{2}=+80^{\circ }$, respectively, with 40 dB interference-to-noise ratios (INRs). For all tested null broadening algorithms, we set up the regions of null broadening to be $\left[-32^{\circ },-28^{\circ }\right]$ and $\left[38^{\circ },42^{\circ }\right]$. In the reconstruction of the INC matrix, the region of the desired signal was set to be located in $\Theta =[2^{\circ },+8^{\circ }]$, and the power parameters were set as $\kappa_{1,2}=1$. In the simulations, the interval of the virtual interference sources was set as $\Delta \eta =0.5^{\circ }$, which meant that there were eight virtual interferers in the vicinity of each interfering source.

**Figure 2:**
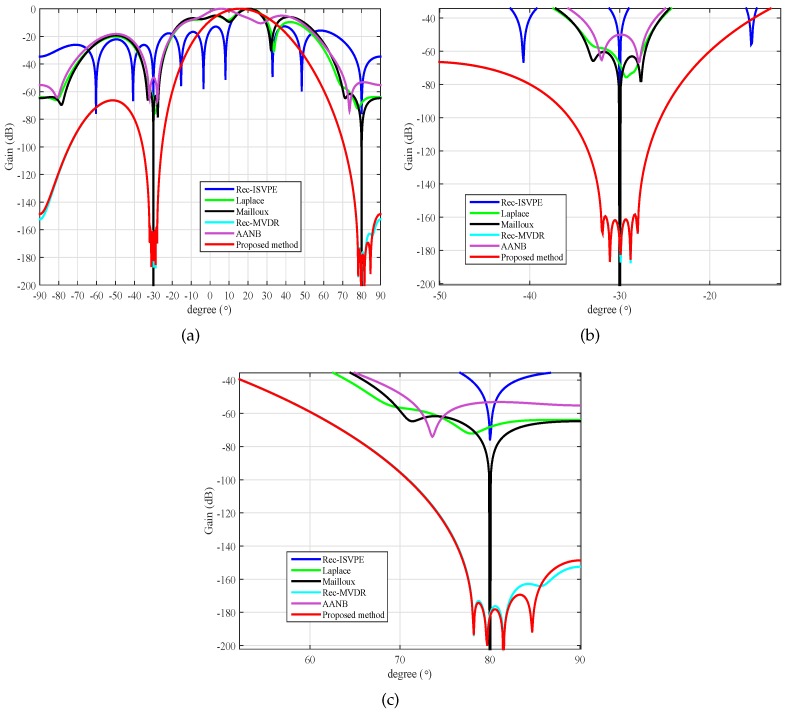
Beampattern of null broadening: (**a**) Array pattern at $\theta_{1}=-30^{\circ }$; (**b**) Null broadening at $\theta_{1}$; and (**c**) Null broadening at $\theta_{2}=+40^{\circ }$.

Figure 2a demonstrates, from the array beampatterns, that null broadening was achieved. Compared to the Rec-ISVPE beamformer, which is a robust and adaptive beamforming technique without wide nulling, the proposed null broadening method could widen the main lobe and raise the sidelobes. As shown in Figure 2b,c, the beampattern of the proposed method was very close to that of Rec-MVDR, and their null depths significantly outperformed the other tested algorithms. In order to better compare the effect of null broadened, the numeric widths of nulls obtained by different algorithms in Figure 2 were measured, with the results reported in Table 1. These numeric widths were the averages of the two nulls at $-30^{\circ }$ and $80^{\circ }$. The results of Table 1 demonstrated that the proposed and Rec-MVDR methods could effectively broaden the nulls low to the beampattern gain at −150 dB, with the null widths similar to the target width $4^{\circ }$, while other tested beamformers could not. Moreover, the null at angle $80^{\circ }$ was close to the end of the array beampattern, which caused a small degree of distortion of the beampattern, and the lowest beampattern gain of the nulls was below −150 dB, where the actual width was a little smaller than $4.2^{\circ }$ and more similar to $4^{\circ }$. Furthermore, the beampattern of Rec-MVDR also confirmed these analyses. Therefore, the proposed method demonstrated good performance of null broadening.

### 4.2. Interference Suppression of Fast-Moving Jammers

In the second example, we examined the performance of the considered methods in suppressing fast-moving interfering signals. The directions of the two interfering signals were assumed to be random and uniformly distributed in $\left[-32^{\circ },-28^{\circ }\right]$ and $\left[38^{\circ },42^{\circ }\right]$, respectively, while the desired signal impinged upon the array from the angular sector $\left[8^{\circ },12^{\circ }\right]$. Moreover, the DOAs of the interferers varied in each trial, remaining fixed from snapshot to snapshot. In the performance comparison of output SINR versus input SNR, the input INRs and the number of snapshots were fixed as 40 dB and 60, respectively. In the performance comparison of output SINR versus the number of snapshots, the input SNR and INR were set as 10 dB and 40 dB, respectively.

**Figure 3:**
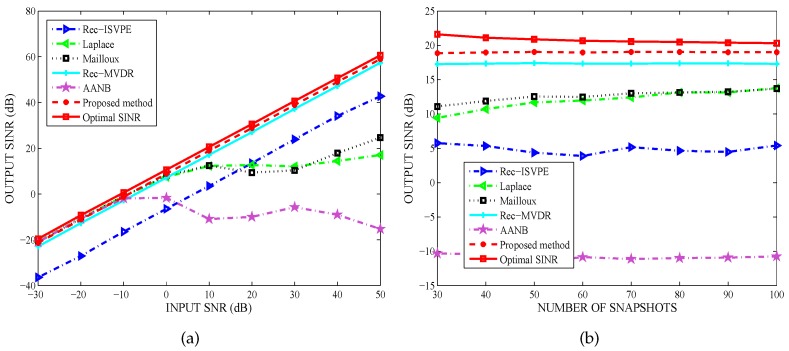
Interference Suppression: (**a**) Output signal-to-interference-plus-noise ratio (SINR) versus input signal-to-noise ratio (SNR); and (**b**) Output SINR versus snapshot number.

Figure 3a shows that the performance of the proposed method was very close to the optimal SINR, while the input SNRs varied in a large range (i.e., from −30 dB to 50 dB). As shown from Figure 3b, the number of snapshots had no effect on the output SINR, and the performance of the proposed method was obviously superior to the others. Therefore, when interference motion leads to mismatches between the DOAs and the adaptive nulls, the proposed null broadening beamformer can effectively suppress interference.

### 4.3. Mismatch Due to Signal Look-Direction Error

In the third example, the impact of the signal look-direction error on the output SINR was examined. We assumed that the random DOA mismatches of the desired signal were uniformly distributed in $\left[-4^{\circ },4^{\circ }\right]$, and the motion of the strong interferers was the same as in the previous simulation. Note that the random DOAs of the desired and interference signals changed from trial to trial, but remained fixed from snapshot to snapshot. When examining the effect of the input SINR, the input INRs and the number of snapshots were fixed as 40 dB and 80, respectively. The input SNR was set to 10 dB in the performance comparison of output SINR versus the number of snapshots.

**Figure 4:**
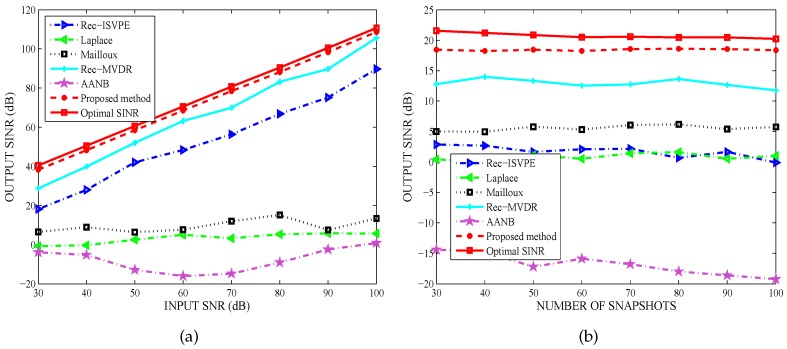
Performance against direction of arrival (DOA) error: (**a**) Output SINR versus input SNR; and (**b**) Output SINR versus snapshot number.

Figure 4a compares the output SINR versus the input SNR. It can been seen that the performance of the proposed algorithm was better than that of the other beamformers and was close to the optimal SINR. In Figure 4b, the output SINR versus the number of snapshots is illustrated. The number of snapshots had no obvious effect on the performance of all tested beamformers. Thus, it has been demonstrated that the performance of the proposed beamformer against DOA errors is obviously better than that of Rec-MVDR. Compared to the remaining beamformers, the proposed algorithm displayed significantly better performance.

### 4.4. Mismatch Due to Array Geometry Errors

In the fourth example, the effects of array geometry mismatches on the tested beamformers was investigated. Mismatches in antenna geometry are usually modelled by sensor position errors, which can be assumed to be subject to a Gaussian distribution. In the simulations, the mismatches were modelled as a Gaussian process with a mean of 0 and a variance of 0.002. At the same time, the DOAs of the interferers changed from run to run, but were kept fixed from snapshot to snapshot. In addition, the other parameters were the same as those in the third example.

**Figure 5:**
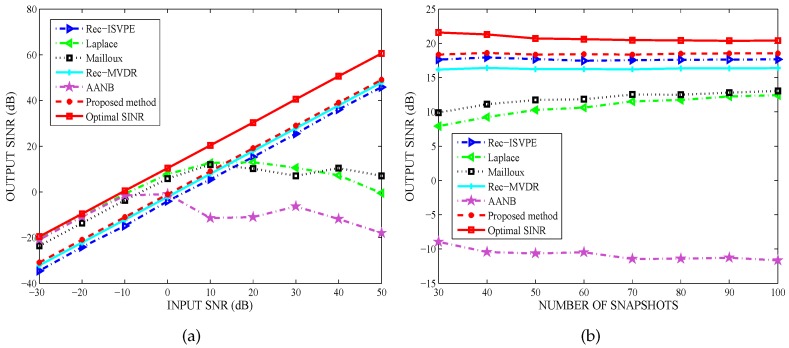
Performance against array geometry errors: (**a**) Output SINR versus input SNR; and (**b**) Output SINR versus snapshot number.

From Figure 5a, it can be seen that the performance of the Laplace- and Mailloux-based methods was superior to that of the tested null broadening algorithms at low SNRs, but dramatically degraded with an increase of input SNR. For the proposed beamformer, array geometry mismatches hardly degraded the output SINR. From Figure 5a, at high SNRs, the proposed beamformer still outperformed the other tested methods. Figure 5b illustrates that the number of snapshots had no or very little effect on the performance of the tested beamformers. Therefore, due to array geometry errors, the performance of the proposed beamformer declined, to a certain degree, but its overall performance was still acceptable.

### 4.5. Mismatch Due to Incoherent Local Scattering

In the fifth example, the impact of the steering vector mismatch due to incoherent scattering was considered. Assuming that the steering vector of the desired signal changes over time, the desired signal can be modeled as [3]

(23) $${\tilde{x}}_{s}\left(k\right)=x_{s}\left(k\right)a(\theta_{s})+\sum_{p=1}^{4}s_{p}^{\prime }\left(k\right)a^{\prime }\left(\theta_{p}^{\prime }\right),$$

where a denotes the steering vector of the desired signal corresponding to the direct angle $\theta_{s}$, $a^{\prime }\left(\theta_{p}^{\prime }\right),p=1,2,3,4$ denote the steering vectors of the incoherently scattered signals, the directions $\theta_{p}^{\prime },p=1,2,3,4$ are independently distributed by a Gaussian distribution with mean $\theta_{s}=10^{\circ }$ and standard deviation $2^{\circ }$ in each run of the simulation, and $s_{p}^{\prime }\left(k\right),p=1,2,3,4$ are independently and identically distributed complex random variables drawn from a random generator rand(0,1). In these scenarios, the signal covariance matrix $R_{s}$ is no longer a rank-one matrix, and the output SINR should be rewritten as [35]

(24) $${SINR}_{opt}=\frac{w^{H}R_{s}w}{w^{H}R_{\mathrm{int}+n}w},$$

which can be maximized by [35]

(25) $$w_{opt}=P\left\{R_{\mathrm{int}+n}^{-1}R_{s}\right\},$$

where P{·} stands for the principal eigenvector of a matrix, which is the eigenvector corresponding to the maximal eigenvalue. The DOAs $\vartheta_{1}\;\mathrm{and}\;\vartheta_{2}$ of the two interferers are uniformly distributed in $\left[-32^{\circ },-28^{\circ }\right]$ and $\left[38^{\circ },42^{\circ }\right]$, respectively. In addition, it must be noted that $\vartheta_{1}$, $\vartheta_{2}$, and $\theta_{p}^{\prime },p=1,2,3,4$ change from run to run, but are kept fixed from snapshot to snapshot.

**Figure 6:**
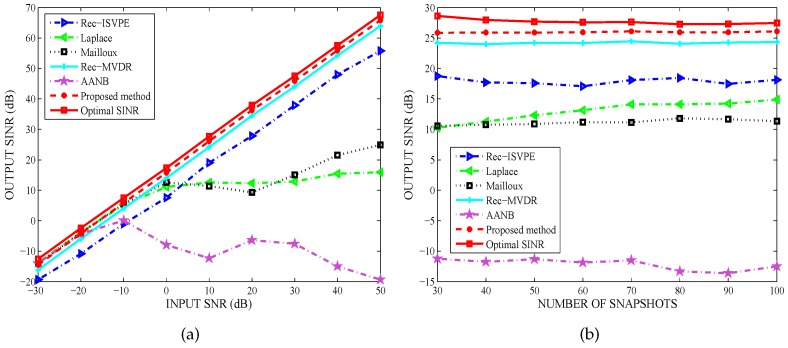
Performance against Incoherent Local Scattering: (**a**) Output SINR versus input SNR; and (**b**) Output SINR versus snapshot number.

It can be seen, from Figure 6a, that the output SINR of the proposed beamformer was very close to the optimal SINR and its robustness against incoherent local scattering was better than the other tested methods. Figure 6b indicates the output SINR versus number of snapshots, which did not affect the performance of any of the algorithms. In general, the proposed algorithm has good robustness against mismatching due to incoherent local scattering.

### 4.6. Performance Comparison against Multiple Interferers

In the last example, the performance against multiple interferers is examined. Assumed that five interfering sources with random waveforms were uniformly distributed in $\left[-62^{\circ },-58^{\circ }\right]$, [$-37^{\circ },-33^{\circ }]$, $\left[17^{\circ },23^{\circ }\right]$, $\left[37^{\circ },43^{\circ }\right]$, $\left[52^{\circ },58^{\circ }\right]$, respectively, and the INRs of five interferers were set as 40 dB, 20 dB, 25 dB, 30 dB, 35 dB, respectively. The desired signal with input SNR = 10 dB came from the direction $-15^{\circ }$. The weight vectors of all tested beamformers could be obtained by broadening the nulls at the central angles.

**Figure 7:**
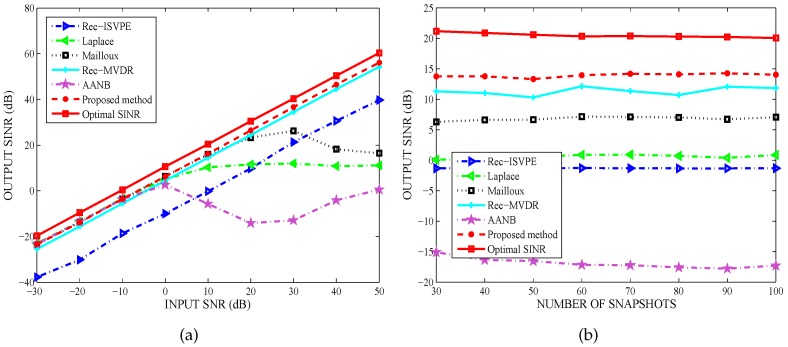
Performance comparsion against multiple interferers: (**a**) Output SINR versus input SNR; and (**b**) Output SINR versus snapshot number.

The output SINR of the tested beamformers versus input SNR for the number of snapshots K=60 was shown in Figure 7a, while Figure 7b compared the output SINR versus the number of snapshots at a SNR of 10 dB. The simulation results showed that the output SINRs of all tested algorithms decreased in the case of multiple interferers. However, the proposed beamformer effectively suppressed multiple interferers and obviously outperformed the other tested algorithms. It has been demonstrated that the number of interferers has little impact on the anti-jamming performance of the tested beamformers, and the proposed null broadening beamforming technique is robust against multiple interferers with different INRs.

## 5. Conclusions

The paper proposes a novel robust beamforming technique using null broadening for mitigating intereference caused by fast-moving jammers or antenna platform motion. The weight vector can be obtained by introducing the reconstructed INC matrix and the estimated the steering vector of the desired signal into the traditional MVDR beamformer. The DOAs of interfering signals can be estimated with the Capon spectrum estimator, and a new INC matrix can be reconstructed by placing several virtual interference sources around each of the actual jammers. The null depth can be controlled by the spatial power parameter in the process of reconstruction. Using convex optimization software, the steering vector of the desired signal can be estimated by solving a new optimization problem based on the reconstructed INC and SNC matrices. The simulation results demonstrated that the performance of the proposed null broadening beamformer dramatically outperforms other methods including classic robust adaptive beamforming and null broadening techniques, and can achieve good robustness against fast-moving jammers or antenna platform motion. Moreover, if the DOAs of the interferers or the direction of antenna platform motion is known, more virtual interference sources can be set along the actual interference DOAs, in order to achieve better performance.

## Figures and Tables

**Table 1:** Null widths at different beampattern gains.

	Beampattern Gain		
Beamformer	−50 dB	−100 dB	−150 dB
Rec-ISVPE	$2.4^{\circ }$	<0.1${}^{\circ }$	<0.1${}^{\circ }$
Laplace	$6.9^{\circ }$	<0.1${}^{\circ }$	can not reach
Mailloux	$6.4^{\circ }$	<0.1${}^{\circ }$	<0.1${}^{\circ }$
Rec-MVDR	$15.2^{\circ }$	$6.9^{\circ }$	$4.2^{\circ }$
AANB	$5.8^{\circ }$	can not reach	can not reach
Proposed method	$15.1^{\circ }$	$6.9^{\circ }$	$4.2^{\circ }$
